# Quantitative study of GaAs nanowires catalyzed by Au film of different thicknesses

**DOI:** 10.1186/1556-276X-7-589

**Published:** 2012-10-24

**Authors:** Hong-yi Xu, Ya-nan Guo, Wen Sun, Zhi-ming Liao, Timothy Burgess, Hao-feng Lu, Qiang Gao, Hark Hoe Tan, Chennupati Jagadish, Jin Zou

**Affiliations:** 1 Materials Engineering, The University of Queensland, Brisbane, Queensland, 4072, Australia; 2 Department of Electronic Materials Engineering, Research School of Physics and Engineering, Australian National University, Canberra, Australian Capital Territory, 0200, Australia; 3 Centre for Microscopy and Microanalysis, The University of Queensland, Brisbane, Queensland, 4072, Australia

**Keywords:** III-V semiconductor, Electron microscopy, Epitaxial growth, GaAs nanowires, MOCVD

## Abstract

In this letter, we quantitatively investigated epitaxial GaAs nanowires catalyzed by thin Au films of different thicknesses on GaAs (111)_B_ substrates in a metal-organic chemical vapor deposition reactor. Prior to nanowire growth, the de-wetting of Au thin films to form Au nanoparticles on GaAs (111)_B_ in AsH_3_ ambient at different temperatures is investigated. It is found that with increasing film thickness, the size of the Au nanoparticles increases while the density of the nanoparticles reduces. Furthermore, higher annealing temperature produces larger Au nanoparticles for a fixed film thickness. As expected, the diameters and densities of the as-grown GaAs nanowires catalyzed by these thin Au films reflect these trends.

## Background

III-V semiconductor nanowires have shown superior electrical and optoelectronic properties due to the reduction of dimension, tunability of their direct bandgaps, and the capability of the bottom-up assembly. These properties make nanowires promising building blocks for future devices and systems. Various nanowire-based applications have already been developed including lasers [1,2], solar cells [3,4], biological and chemical sensors [5], and integrated electronic devices [6]. In order to realize the exceptional properties promised by III-V nanowires, the precise control over the morphologies and structures of the nanowires is the key, which has been devoted with much effort by tuning the growth parameters [7-9]. In general, Au colloidal particles [10], aerosols, and/or thin films [11] can be used as catalysts to induce the epitaxial growth of nanowires in various growth apparatus, including molecular beam epitaxy and metal-organic chemical vapor deposition (MOCVD), which are the most advantageous ones in terms of the precise control of the bottom-up growth of epitaxial III-V nanowires. Among the various catalyst materials and growth apparatus, the use of annealed thin Au films as catalysts in a MOCVD system is a cost-effective and simple approach to induce the growth of III-V nanowires. However, there are only a handful of studies demonstrating the III-V nanowires catalyzed by Au films [11-14]. A number of studies have been focused on the Au film annealing and de-wetting on Si and SiO_2_ substrates [15-17], where the thickness of the film, the annealing temperature, and the morphology of the substrate surface play an important role in controlling the shape and size of the Au particles. In this study, we explore the morphologies of Au thin films deposited on the GaAs (111)_B_ substrates and, more importantly, the effects of the thickness of Au films and the annealing temperature in the formation of Au nanoparticles, when using Au films to grow epitaxial GaAs nanowires. Furthermore, the morphological and structural characteristics of the GaAs nanowires catalyzed by these different thin Au films have also been studied in detail by scanning electron microscopy (SEM) and transmission electron microscopy (TEM). This quantitative study systematically examines the process of film de-wetting to form Au nanoparticles on GaAs(111)_B_ substrates under different conditions and the GaAs nanowires catalyzed by these resulting Au nanoparticles.

## Methods

The GaAs nanowires were epitaxially grown by MOCVD (AIXTRON 200/4, AIXTRON, Herzogenrath, Germany) using trimethylgallium (TMGa) as the group III precursor and arsine (AsH_3_) as the group V precursor. Before growths, Au thin films with different thicknesses (namely, 0.5, 1, 2, 3, and 5 nm, respectively) were deposited onto three batches of GaAs (111)_B_ substrates using an electron beam evaporator under high vacuum (<4×10^−6^ Torr). The thickness of the Au film is measured by a carefully calibrated single-crystal sensor in the evaporator chamber. For each batch, substrates with different thicknesses of Au films were then loaded into a MOCVD reactor simultaneously to ensure the identical growth conditions for the purpose of direct comparison. Firstly, two annealing experiments were carried out at 600°C and 650°C for 10 min in AsH_3_ ambient for two batches of Au-coated (111)_B_ substrates, respectively. For the third batch, the growth of GaAs nanowires was carried out under a pressure of 100 mbar and a total input gas flow rate of 15 slm. Prior to the nanowire growth, the substrates of the third batch were annealed in the growth chamber at 600°C for 10 min under AsH_3_ ambient. The annealing temperature of 600°C was chosen based on the results from the first two annealing batches. The growth temperature of the nanowires was set at 450°C. The flow rates of TMGa and AsH_3_ were set at 1.16 × 10^−5^ mol/min and 5.36 × 10^−4^ mol/min, respectively, resulting in an overall V/III ratio of 46. The nanowire growth parameters were chosen based on the optimal growth condition for growing near defect-free zinc-blende GaAs nanowires catalyzed by Au colloidal particles [12].

The as-grown nanowires were analyzed by SEM (JEOL 7800F, operated at 1.5 kV with an in-lens electron detector setup and at 15kV with a conventional setup; JEOL Ltd., Akishima, Tokyo, Japan) and TEM (Philips Tecnai F20, operated at 200 kV and equipped with energy dispersive spectroscopy (EDS) for compositional analysis; Philips & Co., Eindhoven, The Netherlands). SEM was used to explore the morphologies of the Au thin films of different thicknesses deposited on the GaAs (111)_B_ substrates before and after annealing at different temperatures. Furthermore, morphological characteristics of the as-grown GaAs nanowires, such as their diameters, heights, and densities, are determined by SEM. TEM was used to understand the structural and chemical characteristics of the as-grown nanowires. For TEM investigations, individual nanowires were deposited on holey carbon supporting films. The composition of the post-growth Au catalysts was analyzed by EDS in the scanning TEM mode.

## Results and discussions

**Figure 1:**
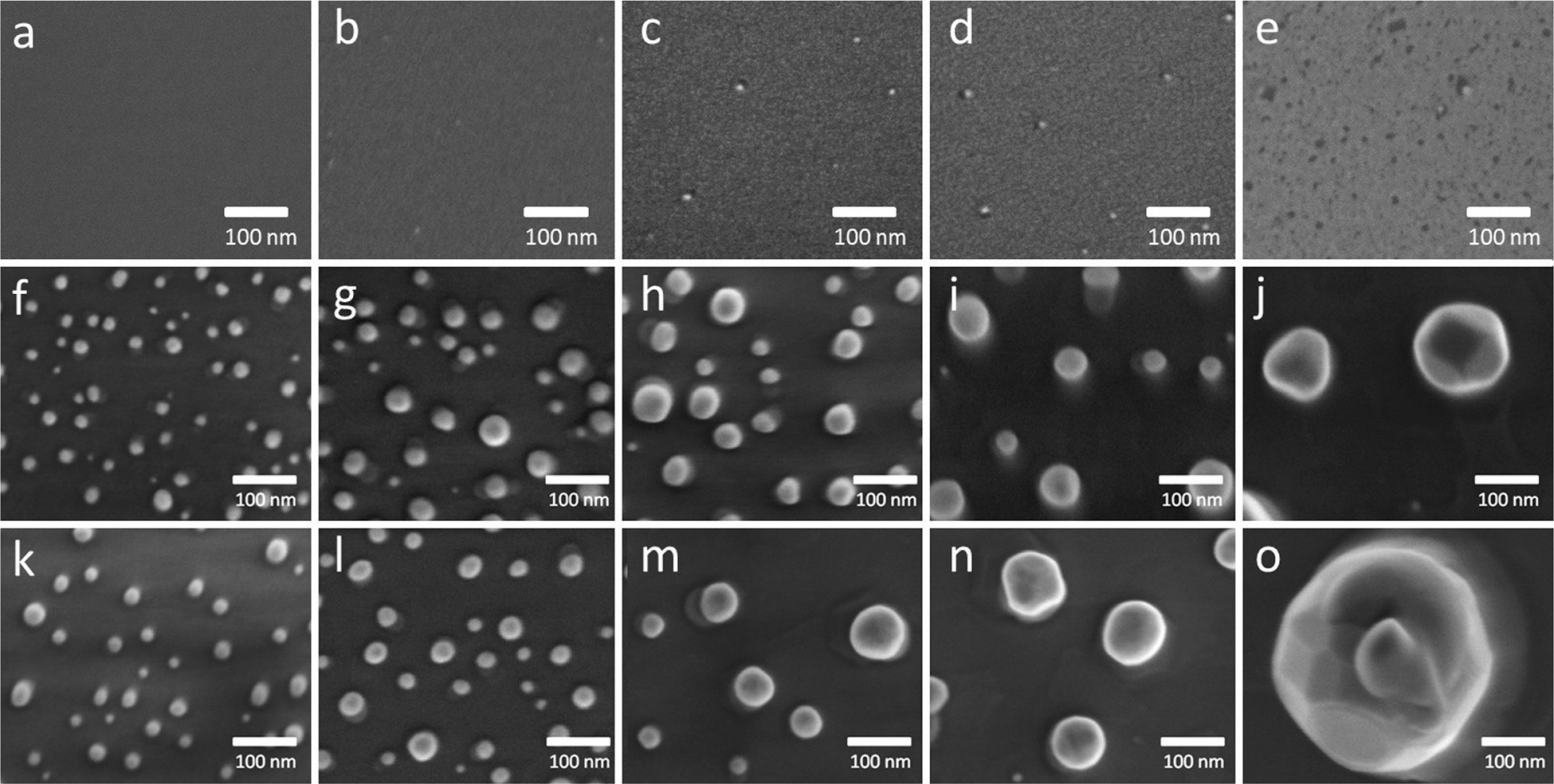
**SEM images of as-deposited and annealed Au thin films of different thicknesses on GaAs(111)_B_ substrates. **(**a** to **e**) Substrates with as-deposited Au thin films. (**f** to **j**) Substrates with Au thin film annealed at 600°C for 10 min. (**k** to **o**) Substrates with Au thin film annealed at 650°C for 10 min. The thicknesses of the Au thin films are 0.5, 1, 2, 3, and 5nm for (**a** to **e**), (**f **to **j**), and (**k** to **o**), respectively.

In order to determine the most suitable annealing conditions for GaAs nanowire growth, we studied the de-wetting processes of the thin Au films with different thicknesses under different annealing temperatures in a quantitative manner. Figure 1 shows plan-view SEM images to demonstrate the typical as-deposited Au thin films and Au nanoparticles produced from the two annealing processes. Figure 1a,b,c,d,e shows the as-deposited Au thin films (0.5, 1, 2, 3, and 5 nm, respectively). In order to obtain superior surface details, the acceleration voltage of the SEM is set at 1.5 keV to minimize the interaction volume of the electron beam in the substrate. It can be seen that, in general, the Au thin film consists of ultra-small nanoparticles evenly covering the GaAs substrates. Occasionally, relatively larger particles of sizes <5 nm can be found on the substrate (the larger and brighter spots visible on the substrates). Figure 1f,g,h,i,j is the SEM images of the Au thin films annealed at 600°C. With increasing the film thickness, the average size of the Au nanoparticles increases. Interestingly, the density of the Au particles is inversely proportional to the thickness of the film. Although similar de-wetting phenomena have been found for Au thin films annealed at 650°C, the average sizes of the Au nanoparticles annealed at this temperature are larger than those annealed at 600°C (Figure 1k,l,m,n,o). According to the Au-Ga phase diagram, our annealing temperatures are well below the melting temperature of pure Au but above the eutectic temperature of the Au-Ga alloy (approximately 340°C) [18]. Therefore, the formation mechanism of the Au nanoparticles follows several steps: (1) At the annealing temperature, the film breaks up and reshapes into irregularly interconnected nanoparticles. Simultaneously, Ga atoms from the substrate start to diffuse into these Au nanoparticles through surface diffusion which rapidly drops the melting temperature of the Au-Ga nanoparticles. (2) By continuing to absorb Ga atoms, the alloy Au-Ga nanoparticles turn into droplets when the Ga concentration in Au particles is beyond the liquidus line. Meanwhile, As atoms are released from the substrates and exhausted out of the chamber with H_2_ carrier gas and excessive AsH_3_ (As atoms). (3) While the substrate is cooled down to room temperature, alloyed Au-Ga nanoparticles are formed. Due to the fact that the cooling down of the MOCVD chamber is under AsH_3_ ambient, it is possible for the Ga atoms in the Au-Ga particles to diffuse out and form a few GaAs epilayers underneath the Au nanoparticles [19]. It is of interest to note that smaller nanoparticles are spherical in shape (Figure 1f,g,k,l), suggesting that the nanoparticles were in the liquid form during the annealing process. Furthermore, we note that, by increasing the annealing temperature, the diffusion rate of the Ga atoms into the Au nanoparticles and the Ostwald ripening effect on the Au thin film (joining of small neighboring nanoparticles to form relatively larger nanoparticles) are both increased [20-22]. As a consequence, when annealing at 600°C, the size of the Au catalysts is smaller and more uniform in size than those formed when annealing at 650°C. Hence, from this study, it is determined that 600°C is the preferred annealing temperature for growing GaAs nanowires using Au thin films on GaAs(111)B substrates.

**Figure 2:**
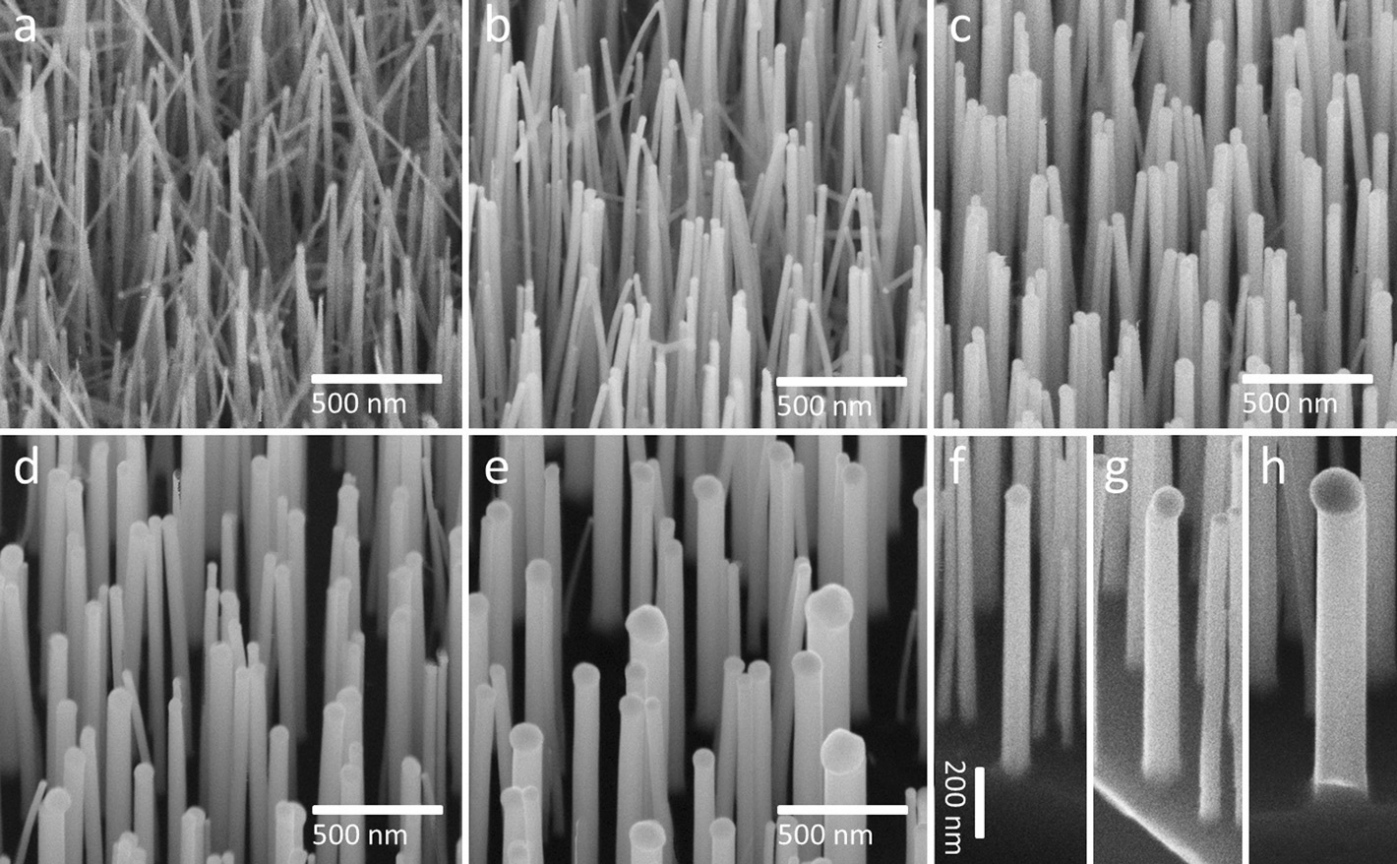
**GaAs nanowires catalyzed by Au thin film of different thicknesses.** The substrates are tilted 30° to give better views of the nanowires. (**a** to **e**) GaAs nanowire grown with 0.5-, 1-, 2-, 3-, and 5-nm Au thin films, respectively. (**f** to **h**) Individual views of GaAs nanowire catalyzed by 2-, 3-, and 5-nm Au thin films, respectively.

After the annealing study, GaAs nanowires were grown using different thin Au films with pre-growth annealing set at 600°C. It should be noted that the growth conditions used here are designed for the growth of perfect zinc-blende-structured GaAs nanowires based on our previous study [7]. As can be seen from Figure 2a,b,c,d,e, the diameters of the nanowires are consistent with the sizes of the de-wetted Au nanoparticles from earlier annealing results. The GaAs nanowires induced by the 0.5-nm-thick Au film are ultrathin and high in density. A small number of kinked nanowires (non-vertically aligned nanowires) were also found among the vertically freestanding nanowires, as shown in Figure 2a. It has been previously reported that when the nanowire diameter decreases below 20 nm, the growth direction of thin nanowires can vary to a non-<111> direction [23,24]. For the thicker film (>2 nm), all nanowires grew vertically. It should be noted that the Au catalysts on the tips of neighboring nanowires attract towards each other due to an artificial effect in SEM, possibly caused by the charge from the electron beam when viewed in the SEM [25,26]. Furthermore, despite the differences in diameter and density, the GaAs nanowires catalyzed by the 2-, 3-, and 5-nm films are similar in length (refer to Figure 2f,g,h).

**Figure 3:**
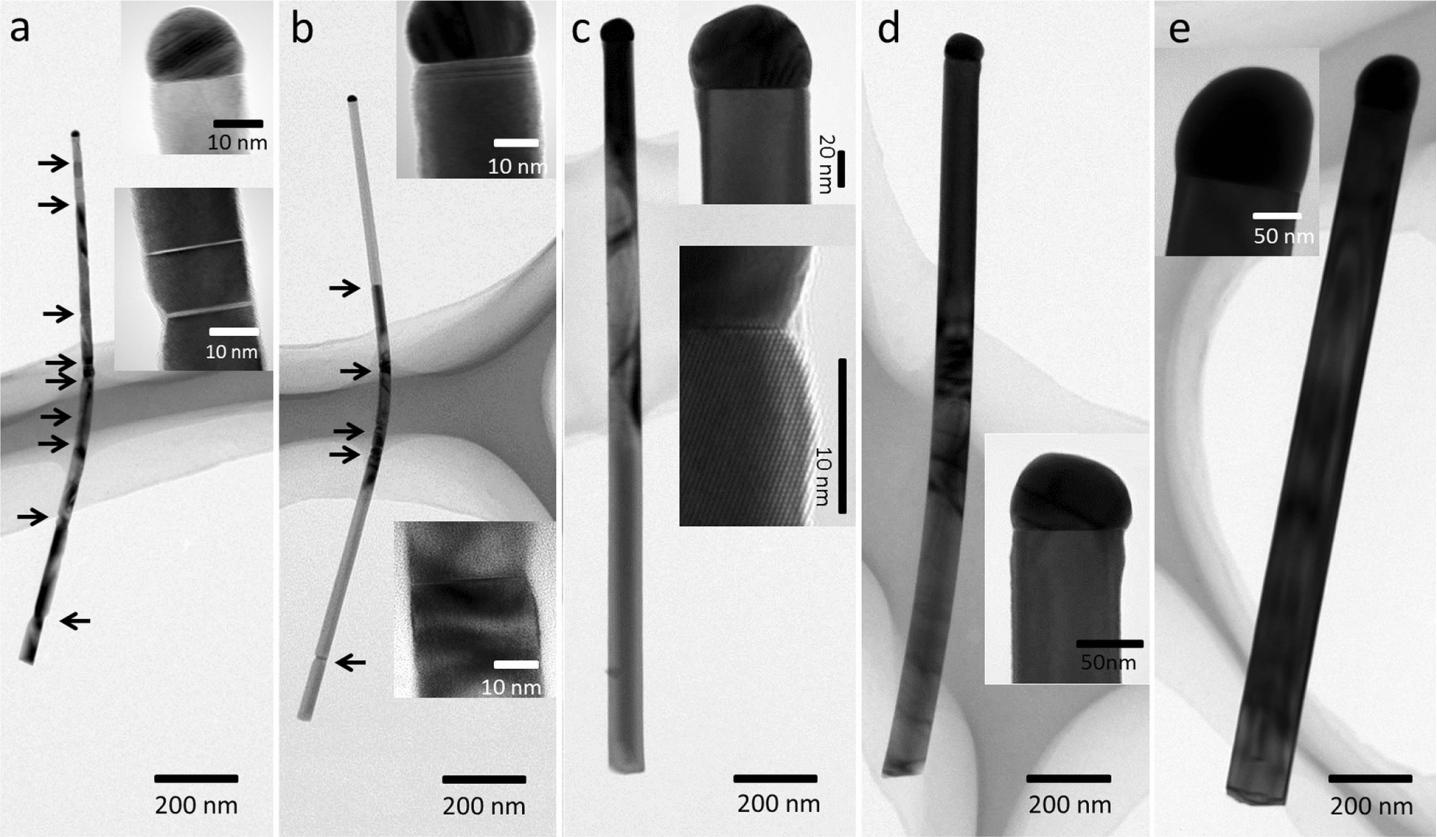
**TEM images of the GaAs nanowires catalyzed by Au thin films of different thicknesses.** The thicknesses of the Au thin films are (**a**) 0.5, (**b**) 1, (**c**) 2, (**d**) 3, and (**e**) 5nm. The two insets in (**a**), (**b**), and (**c**) are the enlarged views of the region containing the catalyst and the nanowire, and the region containing only the nanowire. The insets in (**d**) and (**e**) are the enlarged views of the regions that contain both the catalyst and the nanowire.

In order to study the crystal structure and compare the crystal quality of the GaAs nanowires, comparative TEM investigations have been carried out on a number of nanowires catalyzed by each of the Au films. Figure 3 shows that the average diameter of the nanowires increases with increasing film thickness. Furthermore, as shown by the insets of high-resolution TEM images (Figure 3a,b,c,d,e), we confirmed that all of the nanowires are zinc blende. In fact, the GaAs nanowires catalyzed by thicker Au thin films (>2 nm) are defect-free over their entire length, with the nanowire diameter ranging from 40 to 65 nm. In the case of thinner Au thin films (<1 nm), stacking faults and twins are found, where the diameters of those nanowires were measured to be less than 30 nm. Moreover, with decreasing the nanowire diameters, the density of the planar defects increases as seen in Figure 3a,b (marked by arrows). In vapor–liquid-solid nanowire growth, it has been found that the nucleation of atomic layers of nanowires generally occurs preferentially at the triple-phase line at the catalyst/nanowire interface, where wurtzite and zinc-blende nuclei present some major differences [27]. Depending on the catalyst/nanowire interfacial energies, wurtzite nucleation is favored at high liquid supersaturation in the catalyst, while with the lower supersaturation case, zinc blende is preferred. With smaller catalysts, the supersaturation of Ga atoms increases in the liquid Au-Ga catalyst due to the Gibbs-Thomson effect [28]; hence, short wurtzite sections may be nucleated in the growth of zinc-blende-dominated GaAs nanowires, generating planar faults. It is interesting to note that pure wurtzite and zinc-blende nanowires can be grown by changing only the diameter of the nanowires using nanoparticles of different sizes, with certain growth conditions applied [29,30]. Nevertheless, our study suggests that by using a Au thin film thicker than 2 nm, defect-free zinc-blende GaAs nanowires with a diameter larger than 40 nm can be grown. It is also found that all of the GaAs nanowires grown with different Au films presented in this study have very little tapering (as shown in Figure 3a,e), benefiting from the high growth density of the nanowires [12]. As taper-free and defect-free GaAs nanowires are desired in making optoelectronic devices with excellent properties [10,31], growing GaAs nanowires with a thin Au film demonstrated in this study can be a simple and attractive pathway to achieve the high-quality GaAs nanowires.

**Figure 4:**
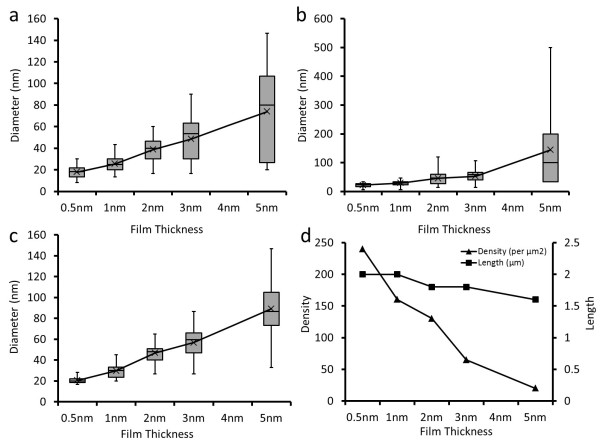
**Quantitative analysis of the effect of the pre-growth annealing on the size/diameter of the nanoparticles/nanowires. **(**a**) The plot of the size of the nanoparticles versus the thickness of Au films, after annealing at 600°C. (**b**) The plot of the size of the nanoparticles versus the thickness of Au films, after annealing at 650°C. (**c**) The plot of the diameter of the GaAs nanowires versus the thickness of Au films. (**d**) The plot of the length and density of the GaAs nanowires versus the thickness of Au films.

In the quantitative study of the growth of GaAs nanowires using thin Au films of different thicknesses, we found that the average size of the Au catalysts created by annealing is linearly proportional to the thickness of the Au thin film prior to the annealing process. Furthermore, by increasing the annealing temperature, the nanoparticle size also increases for a fixed film thickness (refer to Figure 4a,b). As predicted, when catalyzing nanowires with different Au film thicknesses, the diameter of the nanowires is also linearly proportional to the film thickness (Figure 4c). Furthermore, with increasing the film thickness, the density of the GaAs nanowires decreases dramatically, while the average length of the nanowires decreases only slightly (Figure 4d). It is found that 600°C is the optimum annealing temperature for growing a GaAs nanowire using a thin Au film as catalyst. Moreover, when a 2-nm film is used, GaAs nanowires are of high density, defect-free, taper-free, and reasonably uniform in diameter.

## Conclusions

In summary, by the quantitative study of GaAs nanowire growth using Au films of different thicknesses, we found that the Ostwald ripening plays an important role at the pre-growth annealing before the nanowire growth. In the growth of GaAs nanowires with Au film as catalysts, the Ostwald ripening during the annealing process can be enhanced with the increased thickness of the Au film and the annealing temperature. By fine-tuning those two parameters, Au nanoparticles with moderate sizes and narrow distribution in sizes can be produced from Au thin films, which can be used to induce the vapor–liquid-solid growth of high-quality epitaxial GaAs nanowires. This approach provides a promising alternative in the controlled synthesis of III-V nanowires to the approaches using Au nanoparticles.

## Competing interests

The authors declare that they have no competing interests.

## Authors’ contributions

HX carried out the majority of the nanowire growth and characterization. YG, WS, and ZL partially participated in the TEM analysis and discussion. TB, HL, QG, HT, and CJ partially participated in the nanowire synthesis and related supervision. JZ conceived the study. HX, YG, QG, HT, CJ, and JZ wrote this manuscript. All authors read and approved the final manuscript.
